# Use of electronic cigarettes among physicians, residents, and medical students in a referral hospital in the state of Amazonas, Brazil: a cross-sectional study

**DOI:** 10.36416/1806-3756/e20250214

**Published:** 2026-05-22

**Authors:** Luiz Alexandre Silva de Paula Soares, Luísa Tiemi Souza Tuda, Márcio Luís Lombardi Martinez, Roberta Lins Gonçalves

**Affiliations:** 1. Hospital Universitário Getúlio Vargas - HUGV - Universidade Federal do Amazonas - UFAM - Manaus (AM) Brasil.; 2. Universidade Federal do Amazonas - UFAM - Manaus (AM) Brasil.; 3. Universidade do Estado do Amazonas - UEA - Manaus (AM) Brasil.; 4. Programa de Pós-Graduação em Ciências da Reabilitação e Desempenho Físico-Funcional, Universidade Federal de Juiz de Fora - UFJF - Juiz de Fora (MG) Brasil.

**Keywords:** Electronic nicotine delivery systems, Smoking, Students, medical

## Abstract

**Objective:**

To determine the prevalence of electronic cigarette use among physicians, residents, and medical students at a university hospital in northern Brazil.

**Methods:**

We conducted a cross-sectional observational study using a semi-structured questionnaire to collect data on participants’ knowledge and use of electronic cigarettes.

**Results:**

A total of 36 participants were included, most of whom were young female medical students (58.3%) in the 21- to 30-year age bracket. Although 91.7% were aware of electronic cigarettes and their associated health risks, 16 participants reported recreational use of such devices.

**Conclusions:**

Despite high awareness of the potential harms of electronic cigarettes, a considerable proportion of participants-primarily young female medical students-continue to use electronic cigarettes recreationally, highlighting the need for targeted educational and preventive strategies among future health care professionals.

## INTRODUCTION

Electronic cigarettes-also referred to as e-cigarettes, vapes, vaping devices, and electronic nicotine delivery systems (ENDS)-are battery-powered products first introduced in the United States in 2006 and in Brazil three years later as alternatives to conventional cigarettes. ENDS deliver nicotine through the vaporization of a liquid, or “e-liquid,” without combustion, which prolongs use and predominantly attracts adolescents and young adults.^1^ ^-^ ^3^

E-liquids typically contain psychoactive compounds such as nicotine-in the form of either freebase or nicotine salt-solvents such as propylene glycol and vegetable glycerin, and various flavoring agents. The addition of benzoic acid to nicotine salts enables higher nicotine concentrations, markedly increasing the addictive potential of ENDS.^4^ ^-^ ^6^

Although marketed as a less harmful alternative to traditional tobacco, e-cigarettes have been linked to significant health risks, including the e-cigarette or vaping product use-associated lung injury outbreak, with 2,807 cases and 68 deaths in the United States in 2020.^7^ Cases of e-cigarette or vaping product use-associated lung injury have been associated with diffuse alveolar damage and acute fibrinous pneumonitis, although the precise pathophysiological mechanisms remain incompletely understood.^8^ ^-^ ^11^

In the United States, electronic cigarettes rapidly gained popularity among adolescents, becoming the most commonly used tobacco product among this group as early as 2014. By 2020, approximately 3.6 million middle and high school students reported using e-cigarettes in the previous 30 days, with more than 80% indicating a preference for flavored products.^4^ ^,^ ^5^ In Brazil, similar concerns have emerged more recently. 

In northern Brazil, including the state of Amazonas, the use of electronic cigarettes has become an increasingly significant public health concern. Earlier data already indicated emerging use among adolescents, particularly when considering lifetime experimentation. According to the 2019 Brazilian National School-based Adolescent Health Survey, 12.3% (95% CI, 11.1-13.4) of students in the 13- to 17-year age bracket in northern Brazil reported having used electronic cigarettes at least once in their lifetimes. More recent findings from the 2024 Brazilian National School-based Adolescent Health Survey indicate a substantial increase in this behavior, with 21.5% (95% CI, 19.4-23.7) of adolescents in northern Brazil having reported the use of electronic cigarettes at least once in their lifetimes.^12^ ^,^ ^13^ Complementing these findings, the 2022 *Inquérito Telefônico de Fatores de Risco para Doenças Crônicas Não Transmissíveis em Tempos de Pandemia* (Covitel, Telephone Survey of Risk Factors for Chronic Noncommunicable Diseases during the COVID-19 Pandemic),^14^ a nationwide study encompassing all five regions of Brazil and focusing on individuals ≥ 18 years of age, assessed major risk factors for chronic noncommunicable diseases, including tobacco use. The findings of the Covitel study revealed that 19.7% of young adults in the 18- to 24-year age bracket had experimented with electronic cigarettes, underscoring a substantial increase in use and highlighting the growing public health risks associated with ENDS.^14^

Given the rapid rise in electronic cigarette use and the scarcity of research in northern Brazil, the objective of the present study was to assess the prevalence and characteristics of ENDS use among health care professionals and medical students in the state of Amazonas. The relevance of the present study lies in providing evidence to guide targeted and effective public health interventions, particularly in regions facing substantial health challenges. 

## METHODS

This was a cross-sectional observational study assessing the knowledge and use of electronic cigarettes among health professionals and medical students by means of a semi-structured questionnaire. The study was conducted in 2023 at the Getúlio Vargas University Hospital, which is affiliated with the Federal University of Amazonas, located in the city of Manaus, Brazil. The target population included medical students, resident physicians, and non-medicine residents working at the Getúlio Vargas University Hospital during the study period. 

The eligible population consisted of 656 individuals, including 450 medical students, 156 resident physicians, and 50 non-medicine residents. The sample size was calculated for a 5% significance level and 80% statistical power, resulting in a final sample of 243 participants. Simple stratified sampling was used to ensure proportional representation of each subgroup, resulting in 167 medical students, 58 resident physicians, and 18 non-medicine residents. 

Data were collected using a 56-item questionnaire adapted from previous research,^14^ which assessed socioeconomic and epidemiological characteristics, as well as smoking behavior and knowledge of electronic cigarettes. Permission to use the questionnaire was obtained from the original authors. Descriptive and inferential analyses were conducted, with normality being assessed with the Shapiro-Wilk test. Qualitative variables were presented as absolute and relative frequencies, and associations between risk factors and e-cigarette use were evaluated with the chi-square test or Fisher’s exact test, the level of significance being set at 5%. 

## RESULTS

A total of 36 individuals participated in the study, with women comprising the majority (i.e., 21 [58.3%] of the study participants). Most of the respondents were in the 21- to 30-year age bracket, and medical students represented the largest subgroup, accounting for 25 participants (69.4% of the sample; Table 1). 

**Table 1:** Socioeconomic characteristics of the study participants.

Characteristic	n = 36	%
Sex		
Female	21	58.3
Male	15	41.7
Age range		
19-20 years	4	11.1
21-30 years	20	55.6
> 30 years	2	5.6
Marital status		
Single	30	83.3
Married	4	11.1
Living as married	1	2.8
Other	1	2.8
Religion		
Catholic	16	44.4
Protestant	2	5.6
Spiritualist	2	5.6
Other	3	8.3
No religion	13	36.1
Birthplace		
Manaus	24	66.7
Amazonas countryside	2	5.6
Other	9	25.0
Occupation		
Medical student	25	69.4
Physician	11	30.6
Work shift		
Morning shift	1	2.8
Morning and afternoon shift	7	19.4
Morning and afternoon and night shifts	25	69.4
Afternoon and night shifts	3	8.3
Works in more than one place		
Yes	14	38.9
No	22	61.1
Work sector		
Public	17	47.2
Private	3	8.3
Public and private	2	5.6
Not reported	14	38.9

Most (i.e., 27 [75%] of the study participants) reported having contracted COVID-19. A smaller proportion (i.e., 13 [36.1%] of the study participants) reported that they had family members who smoked (Table 2). 

**Table 2:** Background of the study participants.

Characteristic	n = 36	%
Medication use?		
Yes	13	36.1
No	23	63.9
Respiratory disease		
Yes	6	16.7
No	20	55.6
Which respiratory disease?	(n = 6)	
Asthma	5	83.3
Rhinitis	1	16.7
Had COVID-19?		
Yes	27	75.0
No	5	13.9
Does not know	4	11.1
Physically active?		
Yes	24	66.7
No	12	33.3
Frequency of physical activity	(n = 24)	
Once a week	1	4.2
2 to 3 times a week	13	54.2
4 to 5 times a week	10	41.7
Smokers in the family?		
Yes	13	36.1
No	23	63.9
Which family member smokes?	(n = 13)	
Father	2	15.4
Mother	1	7.7
Sibling	1	7.7
Other	9	69.2

Most (i.e., 33 [91.7%] of the study participants) reported being aware of e-cigarettes and recognized their potential harm to health. Of the total sample, 16 (44.4%) reported using e-cigarettes. Among those, 14 (87.5%) reported that they smoked in the company of friends, and 7 (43.8%) reported primarily using e-cigarettes in public places (Table 3). 

**Table 3:** Participants’ knowledge about electronic cigarettes.

Characteristic	n = 36	%
Do you know about electronic cigarettes?		
Yes	33	91.7
No	3	8.3
Have you tried electronic cigarettes?		
Yes	16	44.4
No	20	55.6
How many times?	(n = 16)	
1-3	9	56.3
> 3	3	18.8
Countless times	4	25.0
Where do you smoke?	(n = 16)	
Public places	7	43.8
College	1	6.3
Friends’ homes	3	18.8
Other places	5	31.3
With whom do you smoke?	(n = 16)	
Friends	14	87.5
Not reported	2	12.5
Are electronic cigarettes harmful to health?		
Yes	33	91.7
Maybe	3	8.3

A borderline positive association was observed between having family members who smoked and e-cigarette use (p = 0.052), although no other statistically significant relationships were identified in the preliminary data (Table 4). 

**Table 4:** Relationship between participants’ background and e-cigarette use.

Background	Use of electronic cigarettes			N = 36		p*
	Yes (n = 16)	%	No (n = 20)	%		
Medication use?						0.877
Yes	6	37.5	7	35.0	13	
No	10	62.5	13	65.0	23	
Respiratory disease?						0.230
Yes	4	25.0	2	10.0	6	
No	12	75.0	18	90.0	30	
Had COVID-19?						0.434
Yes	13	81.3	14	70.0	27	
No/Does not know	3	18.8	6	30.0	9	
Physically active?						0.091
Yes	13	81.3	11	55.0	24	
No	3	18.8	9	45.0	12	
Smokers in the family?						0.052*
Yes	3	18.8	10	50.0	13	
No	13	81.3	10	50.0	23	

*Pearson’s chi-square test/Fisher’s exact test. Values of p < 0.05 (5%) were considered significant.

## DISCUSSION

Preliminary results from our study indicate a higher use of ENDS among young female medical students. This contrasts with most of the existing literature on tobacco use, in which male students typically predominate. For example, in a study conducted in the state of Acre, in northern Brazil, it was reported that male medical students were more likely to smoke,^15^ ^,^ ^16^ and a similar pattern was observed in a study conducted in Nepal.^17^ However, other research highlights geographic variations in smoking patterns among medical students, suggesting that demographic and cultural contexts influence these behaviors.^18^ In Brazil, the increasing participation of women in medical schools may partly explain this trend, although further investigation is needed to understand the social and cultural determinants of ENDS use among this population. 

Consistent with prior studies, our results show that having family members who smoke is positively associated with e-cigarette use.^15^ Participants demonstrated a high level of knowledge regarding the risks of electronic cigarettes, yet recreational use persisted, suggesting a gap between theoretical awareness and practical behavior.^18^ The preference for using e-cigarettes in social settings-such as groups of friends or public spaces-underscores the role of interpersonal interactions and social dynamics in the adoption of ENDS. This behavior may reflect the search for social acceptance and contemporary patterns of leisure, emphasizing the need to consider social and behavioral factors when evaluating e-cigarette use among highly educated young adults. 

Our findings indicate a borderline positive correlation between living with smokers and e-cigarette use (p = 0.052), suggesting family influence as a relevant factor, in line with previous literature highlighting family exposure as a risk factor for tobacco initiation.^15^ ^,^ ^19^ Although no other statistically significant associations were observed-likely due to the small sample size and challenges in engaging health care professionals-continued data collection is essential to increase statistical power and explore additional correlations. Expanding the sample may provide more robust insights into factors influencing ENDS use among this population. 

A notable concern is the discrepancy between participants’ awareness of health risks and continued use of e-cigarettes, particularly among medical students, who are expected to serve as health promoters. This aligns with national data showing a high prevalence of e-cigarette experimentation among young Brazilians in the 15- to 24-year age bracket, who represent the majority of users.^12^ ^-^ ^14^ The 2022 Covitel study further emphasized this trend, highlighting the growing risk among young, educated adults.^14^

These preliminary results have important public health implications. They underscore the urgency of educational campaigns and targeted interventions aimed at future health care professionals, whose credibility in promoting healthy behaviors may be undermined if they themselves engage in risky practices.^20^ Additionally, the limited discussion of e-cigarettes in medical curricula^16^ ^,^ ^18^ ^,^ ^21^ points to a critical gap in professional training. 

In summary, despite limitations such as sample size and recruitment challenges, our findings provide valuable insights into the use of ENDS among health professionals. They highlight the influence of family environment, social dynamics, and demographic shifts on e-cigarette use, as well as the persistent gap between knowledge and behavior. Importantly, the present study contributes evidence from northern Brazil, particularly the state of Amazonas, a region that is largely overlooked in research on electronic cigarette use. These results reinforce the need for educational and preventive strategies tailored to health care students and professionals, and emphasize the importance of expanding research in underrepresented regions to inform evidence-based public policies addressing the growing adoption of alternative tobacco products. 
